# Adaptive Monocular Visual–Inertial SLAM for Real-Time Augmented Reality Applications in Mobile Devices

**DOI:** 10.3390/s17112567

**Published:** 2017-11-07

**Authors:** Jin-Chun Piao, Shin-Dug Kim

**Affiliations:** Department of Computer Science, Yonsei University, 50 Yonsei-ro, Seodaemun-gu, Seoul 03722, Korea; kumcun@yonsei.ac.kr

**Keywords:** monocular simultaneous localization and mapping, visual–inertial odometry, optical flow, adaptive execution, mobile device

## Abstract

Simultaneous localization and mapping (SLAM) is emerging as a prominent issue in computer vision and next-generation core technology for robots, autonomous navigation and augmented reality. In augmented reality applications, fast camera pose estimation and true scale are important. In this paper, we present an adaptive monocular visual–inertial SLAM method for real-time augmented reality applications in mobile devices. First, the SLAM system is implemented based on the visual–inertial odometry method that combines data from a mobile device camera and inertial measurement unit sensor. Second, we present an optical-flow-based fast visual odometry method for real-time camera pose estimation. Finally, an adaptive monocular visual–inertial SLAM is implemented by presenting an adaptive execution module that dynamically selects visual–inertial odometry or optical-flow-based fast visual odometry. Experimental results show that the average translation root-mean-square error of keyframe trajectory is approximately 0.0617 m with the EuRoC dataset. The average tracking time is reduced by 7.8%, 12.9%, and 18.8% when different level-set adaptive policies are applied. Moreover, we conducted experiments with real mobile device sensors, and the results demonstrate the effectiveness of performance improvement using the proposed method.

## 1. Introduction

In recent years, the rapid development of mobile devices such as unmanned aerial vehicles, handhold mobile devices, and augmented reality (AR)/virtual reality (VR) headsets has provided a good platform for AR technology. Simultaneous localization and mapping (SLAM) has become a prominent issue in the field of computer vision and is the key next-generation technology for robots, autonomous driving and AR.

SLAM is a low-level technology that provides map and location information to applications using it. Depending on the application, the requirements for the map and location accuracy are different. For example, if the application target is a robot that performs navigation tasks based on SLAM, it requires the entire map information and perceptible information about obstacles in the surrounding space. This results in a higher demand for SLAM mapping. In AR applications, the real-time camera pose and the distance between the camera and the object are more important, and the accuracy of SLAM system mapping and global positioning is relatively low.

Usually, the computing power and power consumption of mobile devices limit the use of SLAM in mobile devices, since the SLAM algorithm requires higher robustness and computational efficiency. The performance and accuracy, and robustness is a trade-off relationship; hence, in AR applications, in order to obtain a real-time camera pose, the SLAM system must be optimized.

In this paper, we propose an adaptive monocular visual–inertial SLAM for real-time AR applications in mobile devices. It includes modification and implementation based on the monocular ORB-SLAM, which is an oriented FAST and rotated BRIEF (ORB) [1] feature-based monocular SLAM [2]. First, we designed a visual–inertial odometry (VIO) method that combines a camera input and inertial measurement unit (IMU) sensor. By using this method, the distance between the object and the camera and size of the object can be easily calculated, which was used in AR applications to interact between real world and virtual objects. Second, to support real-time faster tracking of AR applications in the mobile device environment, an optical-flow-based fast visual odometry (VO) module was designed and combined with the existing ORB-SLAM system. Finally, we proposed an adaptive execution method that adaptively selects the tracking module according to the change in the IMU sensor value.

We experimentally measured the time cost and accuracy of estimating the camera pose. In order to obtain the optimum performance and accuracy, we experimented with many cases to determine the best threshold for the adaptive SLAM algorithms to balance accuracy with processing speed.

The remainder of this paper is organized as follows. In the following section, we describe the related work on monocular SLAM. In Section 3, we propose an adaptive monocular visual–inertial SLAM, and present four different level-set adaptive policies. In Section 4, we experiment with various cases to demonstrate the performance improvements. Finally, we provide our conclusions in Section 5.

## 2. Related Work

The general structure of a visual SLAM is divided into front-end and back-end. The front-end includes the VO [3] module and the mapping module, and the back-end has the optimization module. There may also be an additional loop-closure detection module [4].

- The VO module estimates the approximate 3D camera pose and structure from adjacent images to provide better optimization of the initial value for the back-end. Visual SLAM is divided into feature-based SLAM and appearance-based SLAM depending on whether the feature points are extracted in VO.
- The mapping module creates a map that will be used mainly in SLAM, and can be used for navigation, visualization, and interaction. The map is divided into a metric map and topological map, according to the type of information. The metric map accurately represents the positional relationship of objects, which are usually divided into sparse and dense objects.
- The optimization module estimates the trajectory and map state from noisy data. This can be viewed as a maximum a posteriori problem [5]. SLAM is divided into a filter-based SLAM and graph-optimization-based SLAM according to the optimization method at the back-end [6].
- The loop-closure detection module determines whether the camera arrives at a scene it has captured before. Loop-closure solves the problem of drifting of the estimated positions over time.

Usually, the SLAM system is divided into feature-based SLAM and appearance-based SLAM according to whether the feature points are extracted.

The feature-based SLAM extracts feature points and descriptors for the input image, calculates the camera pose by matching 2D–2D, 2D–3D and 3D–3D feature points, and performs mapping [7,8,9]. If the entire image is processed, the computational burden is too high. Therefore, feature points can store important information of the image and reduce the amount of computation.

In early algorithms, monocular visual SLAM was often implemented as a filter [10,11,12]. In that approach, stores the 3D coordinates of the camera pose and map points in a state vector, and expresses the uncertainty using a probability density function. The average and standard deviation of the last update vector are obtained using an observation model and recursive calculation. However, it has uncertainties owing to computational complexity and linearization.

A filter-based SLAM estimates only the current state information, regardless of the previous state. In contrast, the nonlinear optimization method [13], which has been commonly used recently, estimates the state by using the data of the entire time period. This method is superior to the traditional filter-based method [6] and is currently the most commonly used visual SLAM method. Parallel tracking and mapping (PTAM) [14] is a typical keyframe-based monocular visual SLAM and uses nonlinear optimization. PTAM uses a keyframe-based odometry method that separates tracking and mapping tasks into two independent modules and parallelizes them with threads. In the mapping module, the keyframes are selected sparsely in the mapping module and the map points observed by these keyframes are used for mapping. This module is very efficient and can easily calculate accurate 3D structures and optimize them using bundle adjustment [15]. The tracking module performs camera tracking tasks in the front-end and can quickly calculate the motion of the current frame. This achieves the necessary efficiency for real-time calculations.

ORB-SLAM [2] is a relatively complete keyframe-based monocular SLAM released by Mur-Artal, Montiel and Tardos in 2015. ORB-SLAM is based on PTAM basic architecture. In addition to the tracking and mapping threads, a loop-closure detection [4,16] thread is added. Feature extraction and mapping, sparse map generation, and place recognition are based on ORB feature points [1]. ORB-SLAM computes the value and weight using bags of words (BoW) [17] for all the image feature points, and matches two images using BoW vector values. Experiments using the KITTI dataset [18] demonstrate that the accuracy and robustness of this method are better than that of PTAM. ORB-SLAM is relatively stable and accurate, can be adapted to various environments, such as indoor/outdoor and large/small scale, and can be executed in real time on a PC. They released the code as open source [19], and ORB-SLAM is a good reference for learning and studying SLAM methods. ORB-SLAM supports automatic map initialization, and the keyframe and map point management mechanisms are relatively comprehensive.

Direct SLAM is a particular case of appearance-based SLAM. Direct SLAM can estimate the camera pose directly from the colors of the pixels of two images, without extracting and matching feature points. This ensures better robustness in situations where there are fewer minutiae or blurred images. The dense tracking and mapping DTAM [20] reconstructs the surrounding map information into a dense 3D depth map model. However, since the DTAM restores the dense map for each pixel and applies global optimization, the computational burden is very high. Engel et al. proposed LSD-SLAM [21] and DSO [22] based on the direct method. Compared with DTAM, LSD-SLAM and DSO use fewer pixels, and, since each pixel depth is calculated independently, they are more efficient than DTAM. The direct-method-based SLAM has the following advantages: it does not extract feature points, it can be used even in the case of a small number of feature points or a blurred image, and it can generate depth map. However, it is ineffective for fast motion and changes in grayscale values, and requires higher hardware requirements for the camera.

Monocular visual SLAM is low-cost and easy to implement. However, the monocular camera cannot obtain depth information. Owing to the uncertainty of depth, monocular visual SLAM has the following problems: the need for initialization; uncertainty of scale; and scale drift.

With the development and dissemination of a variety of hardware sensors, SLAM technology is moving toward multi-sensor fusion, which leverages multiple complementary sensors to achieve higher accuracy and robustness. Mobile devices are usually equipped with a variety of sensors, such as a camera, IMU, and GPS. We focus on combining the standard monocular camera and IMU sensor in mobile devices to implement more accurate and stable SLAM systems. The methods for integrating visual information and IMU data are divided into loosely coupled [23,24] and tightly coupled [25,26,27,28] methods, depending on whether image feature information is added to the state vector. The loosely coupled approach usually involves executing visual-based SLAM and inertial-based modules separately and combining the results to estimate the measurements. However, in a loosely coupled method, the monocular SLAM drift problem still exists. In a tightly coupled method, IMU bias can be corrected by adding image feature information to the state vector and the scale of the monocular SLAM can be estimated. This method is more accurate than the conventional single-visual-sensor-based SLAM.

In this study, we used a tightly coupled method to implement a more complete SLAM system. By tightly coupling and combining the standard monocular camera and inertial measurement unit sensor in mobile devices, we implemented a more accurate and stable SLAM system to solve the existing problems for the commercialization of SLAM-based augmented reality applications.

There are still many problems in commercializing SLAM-based AR applications: the various kinds of mobile devices, the rolling shutter problem of mobile device camera sensors, the uncertainty of scale and scale drift of single-camera-based SLAM, the capturing of blurred images by the camera sensor when moving fast, etc. The fusion of the inertial measurement units (IMU) sensor and camera sensor can solve some of these problems. In a previous study, we implemented an AR application based on marker-less object detection in real time on a mobile device [29]. We designed an object-tracking method using IMU sensor data and image data. The tracking method uses a homography matrix [7], owing to mobile device performance limitations. This system shows good robustness to the translation motions of the camera, but it is ineffective against pure rotation. In order to overcome these limitations, we attempt to incorporate SLAM technology to improve the accuracy and robustness.

We analyzed each SLAM system in the current research trends and selected ORB-SLAM, which is a monocular visual SLAM, as a reference model for the AR application environment. ORB-SLAM supports relatively high speed, accuracy, and robustness.

## 3. Methodologies

This section describes the basic structure of the visual–inertial SLAM and demonstrates the method of implementation of the adaptive method for the system.

First, some abbreviations are defined for clear representation. Table 1 lists the abbreviations used in this paper.

**Figure 1:**
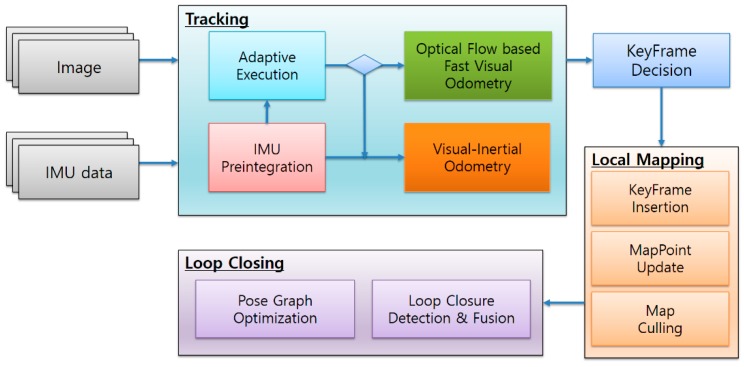
Structural architecture of adaptive visual–inertial simultaneous localization and mapping (SLAM).

The structural architecture of the adaptive visual–inertial SLAM in a mobile device is shown in Figure 1. As shown in the figure, the main architecture includes modules such as tracking, local mapping, and loop closing, consistent with the ORB-SLAM.

In this study, three methodologies are applied in the tracking module.

First, we added the IMU preintegration module to integrate the image data with IMU sensor data, and implemented a visual–inertial integrated VIO method. By using this method, the distance between the object and the camera and size of the object can be easily calculated, which was used in AR applications to interact between real world and virtual objects.

Second, we designed a fast VO method based on optical flow, which estimates the camera pose between the current frame and previous frame. The optical-flow-based fast VO module uses the traced feature points of the existing reference frame without extracting the feature points. Therefore, no additional feature extraction time is required, the execution time of the tracking module can be reduced.

Finally, we proposed an adaptive execution module. In this module, the IMU preintegration value is used to predict the state of motion between the current frame and the previous frame. It is dynamically selected through VIO or optical-flow-based fast VO for current frame tracking according to the state of motion. We also designed adaptive policies at different levels.

Specifically, the algorithm can be simplified to decrease the amount of calculation and resource overhead.

### 3.1. Visual–Inertial Odometry

Cameras and IMU sensors are a common sensor combination in mobile devices. Usually, IMU sensors have much higher frequency than camera sensors. The visual-based VO has high accuracy, but when the camera moves quickly, the image becomes blurred and feature points cannot be extracted or the camera position cannot be tracked. In contrast, the IMU sensor is highly accurate when the camera moves rapidly. Therefore, the VIO method combines the characteristics of these two types of sensors to obtain complementary results.

**Figure 2:**
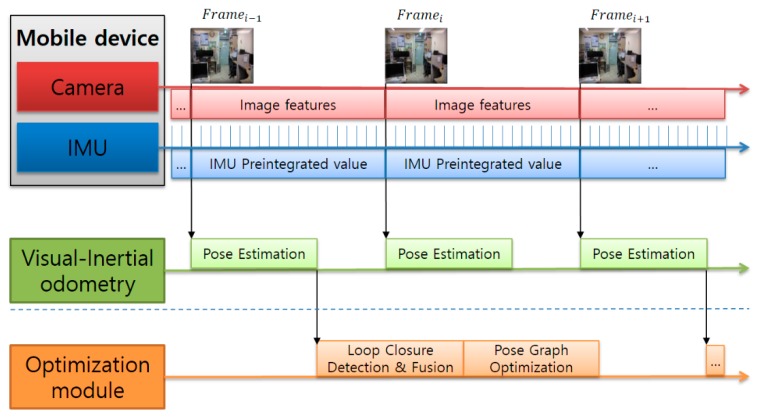
Structure of visual–inertial odometry.

We implemented a real-time VIO method based on the monocular ORB-SLAM. The structure is shown in Figure 2. First, the camera and IMU sensor data are pre-processed through IMU preintegration. The camera pose is estimated using the fused data. In the back-end stage, the loop-closure detection and the pose-graph optimization for the keyframe are performed to optimize the global map. Furthermore, we perform visual–inertial initialization for VIO in the system initialization phase. In the optimization phase, the loop-closure detection and pose-graph optimization modules can be lightly executed in the back-end and switched on/off, depending on the situation.

In general, mobile devices are equipped with cameras and IMU sensors. Therefore, the visual–inertial SLAM can be generally divided into the world coordinate system (World), IMU coordinate system (Body), and camera coordinate system (Camera). There are transformation relations between the coordinate systems. The visual-based systems have coordinate transformations between only the camera and world coordinate systems. The coordinate transformation relation can be expressed as **T**_CW_ = [**R**_CW_|**t**_CW_]. In a visual–inertial SLAM, the connection between the IMU and camera is usually assumed to be rigid. The conversion relation between the IMU and camera coordinate systems can be expressed as **T**_CB_ = [**R**_CB_|**t**_CB_], which can be obtained via calibration in multi-sensor systems [30,31].

#### 3.1.1. IMU Preintegration

The IMU motion model below includes a formula for calculating the subsequent moment state using the current state values and IMU measurement data:

(1) $$\begin{array}{c} R(t+\Delta t)=R(t)\mathrm{Exp}((\tilde{\omega }\left(t\right)-b^{g}\left(t\right)-\eta^{gd}\left(t\right))\Delta t),\\ v(t+\Delta t)=v(t)+g\Delta t+R(t)(\tilde{a}\left(t\right)-b^{a}\left(t\right)-\eta^{ad}\left(t\right))\Delta t\\ p(t+\Delta t)=p(t)+v(t)\Delta t+\frac{1}{2}g\Delta t^{2}+\frac{1}{2}R(t)(\tilde{a}\left(t\right)-b^{a}\left(t\right)-\eta^{ad}\left(t\right))\Delta t^{2}, \end{array}$$

where **R** is the camera rotation, **v** is the motion velocity, **p** is the camera position, **η** is the IMU noise, and **b***^a^* and **b***^g^* are the IMU accelerometer bias and gyroscope bias, respectively. These difference equations explain the constraint relationship between IMU data at two different time points.

In order to incorporate the IMU sensor data into the optimization-method-based SLAM algorithm, IMU information is added as a constraint item to the objective function to be optimized. However, since the IMU sampling rate is very high, the dimension of the variable will be too large if it is optimized for the IMU poses at each time point. Therefore, inertial measurements are usually integrated between frames to constitute relative motion constraints. We used the IMU preintegration [32] method to solve the computational complexity problem of the optimization-method-based visual–inertial SLAM and repeating the integration when the bias estimate changes, and we integrated the IMU preintegration in the original ORB-SLAM.

**Figure 3:**
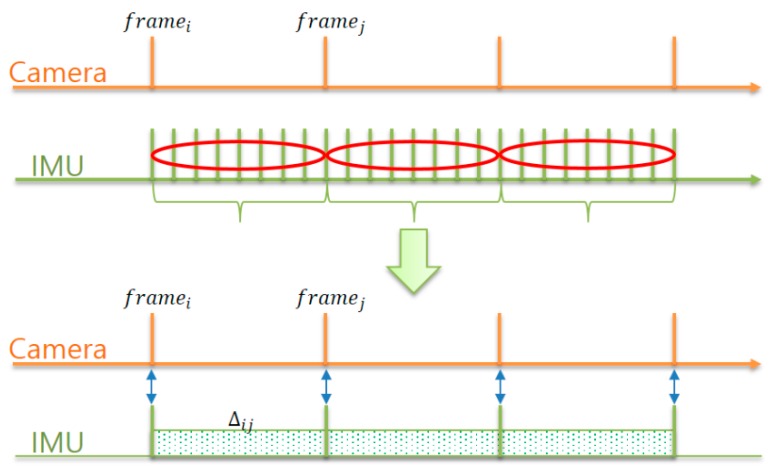
Diagram of the IMU preintegration method.

As shown in Figure 3, the preintegration method integrates all the IMU measurements between two frames *i* and *j* to represent the motion model. The formula is expressed as follows.

(2) $$\begin{array}{c} R_{j}=R_{i}\prod_{k=i}^{j-1}\mathrm{Exp}(({\tilde{\omega }}_{k}-b_{k}^{g}-\eta_{k}^{gd})\Delta t)\\ v_{j}=v_{i}+{g\Delta t}_{ij}+\sum_{k=i}^{j-1}R_{k}({\tilde{a}}_{k}-b_{k}^{a}-\eta_{k}^{ad})\Delta t\\ p_{j}=p_{i}+\sum_{k=i}^{j-1}{[v}_{k}\Delta t+\frac{1}{2}g\Delta t^{2}+\frac{1}{2}R_{k}({\tilde{a}}_{k}-b_{k}^{a}-\eta_{k}^{ad}){\Delta t}^{2}] \end{array}$$

where $\Delta t_{ij}≐\sum_{k=i}^{j-1}\Delta t$ and ${\left(\cdot \right)}_{i}≐\left(\cdot \right)\left(t_{i}\right)$. Equation (2) provides an estimate of the motion between time *t_i_* and *t_j_*.

According to the above formula, the measurement model between the two frames is derived. The following relative motion increments that are independent of the pose and velocity at *t_i_*:

(3) $$\begin{array}{c} \Delta R_{ij}≐R_{i}^{T}R_{j}=\prod_{k=i}^{j-1}\mathrm{Exp}(({\tilde{\omega }}_{k}-b_{k}^{g}-\eta_{k}^{gd})\Delta t)\\ \Delta v_{ij}≐R_{i}^{T}\left(v_{j}-v_{i}-{g\Delta t}_{ij}\right)=\sum_{k=i}^{j-1}R_{ik}({\tilde{a}}_{k}-b_{k}^{a}-\eta_{k}^{ad})\Delta t\\ \Delta p_{ij}≐R_{i}^{T}\left(p_{j}-p_{i}-v_{i}{\Delta t}_{ij}-\frac{1}{2}g\Delta t_{ij}^{2}\right)=\sum_{k=i}^{j-1}[\Delta v_{ik}\Delta t+\frac{1}{2}\Delta R_{ik}({\tilde{a}}_{k}-b_{k}^{a}-\eta_{k}^{ad}){\Delta t}^{2}] \end{array}$$

where $\Delta R_{ik}≐R_{i}^{T}R_{k}$ and $\Delta v_{ik}≐R_{i}^{T}\left(v_{k}-v_{i}-{g\Delta t}_{ik}\right)$. We can calculate the right side of Equation (3) directly from the inertial measurement between the two frames, that is, the relative motion increments between the two frames can be obtained by solving the above equation.

As shown in the above formula in [32], the constraints between the two frames can be expressed using only IMU data; furthermore, they can be expressed according to the states of the two frames. Therefore, we can define the residuals between the observation and state, construct the least-squares solution, and optimize the pose.

**Figure 4:**
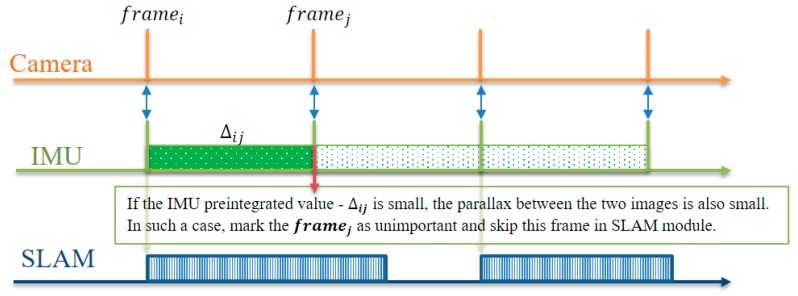
Fusion model of IMU preintegration and the SLAM module. The IMU preintegration value is used to determine the importance of the frame in the SLAM module.

If the IMU preintegration value is small, the camera movement changes only slightly, and the parallax between the two images is also small. Such images are relatively unimportant in the SLAM module. As shown in Figure 4, if the IMU preintegration value −**∆*_ij_*** between the current frame *j* and the previous frame *i* is calculated and this value is less than the threshold value, we mark the current frame *j* as unimportant and subsequently estimate the camera pose using our fast VO module. This frame is not processed by the SLAM module. Using this method, we can select the VO module adaptively at run time.

However, this preprocessing method cannot be applied directly to the monocular ORB-SLAM. This is because the motion between two consecutive frames is assumed to be uniform movement in the monocular ORB-SLAM. In other words, when performing the pose optimization of the current frame, the initial value of the current-frame pose is obtained by using the velocity and pose of the previous frame. If the gap between frames is not constant, this assumption is not accurate, which affects the performance and can even cause the tracking to fail.

(4) $${\mathrm{initialPose}}_{currentFrame}={\mathrm{Velocity}}_{lastFrame}\times {\mathrm{Pose}}_{lastFrame}$$

Visual–inertial SLAM is not affected by this problem because it uses IMU measurements as the initialization data. Therefore, our methodology is only suitable for visual–inertial SLAM.

#### 3.1.2. Initialization

In the IMU preintegration phase, we can estimate the pose from the IMU. However, as shown in the formula below, the IMU measurement data are affected by noise and bias.

(5) $$\tilde{\omega }\left(t\right)=\omega \left(t\right)+b^{g}\left(t\right)+\eta^{gd}\left(t\right)$$

(6) a˜(t)=RWBT(t)(aW(t)−gW)+ba(t)+ηad(t)

In general, noise can be calculated when performing IMU calibration. In the system initialization phase, the IMU accelerometer bias and gyroscope bias should be estimated. The bias is estimated only once in the initialization phase and is not recomputed until the system has changed significantly.

The data measured by the IMU accelerometer can be viewed as the sum of its own acceleration and the earth's gravitational acceleration. Therefore, it is difficult to separate these two accelerations and the error is large. Thus, we proceed with the system initialization using a more accurate gyroscope.

In the initialization phase, we carry out the following steps. First, we estimate the gyroscope bias. With two consecutive keyframes, the initial gyroscope bias can be easily computed and subsequently optimized using the Gauss–Newton method [33]. Subsequently, we update the gyroscope bias and pre-integration values for the keyframes in the local window and approximately estimate the scale of the system and the gravity vector. The scale is the magnification ratio between the system map and the ground-truth, and the gravity vector is a vector of gravity values with a magnitude of approximately 9.8. Finally, we estimate the accelerometer bias using the exact gravity magnitude value as a constraint, and refine the scale and gravity direction.

The IMU biases in the initialization phase are used in the IMU preintegration. The rotation, velocity, and position between two frames can be estimated using Equation (3).

#### 3.1.3. Pose Estimation

In VIO, pose, velocity, and IMU biases can be calculated for every frame. The camera pose of the current frame is predicted using the IMU motion model, and subsequently the map points of the local map are projected onto the current frame and matched with the keypoints of the current frame. The pose is optimized for the current frame by minimizing the projection error of all the matched feature points and the IMU error.

In order to estimate the pose, the state variable is first determined. In tightly coupled visual–inertial SLAMs, the target state value is often used to estimate values such as pose, velocity and IMU biases. The state variable is a 15-dimensional value and is defined as follows:

(7) $$X_{i}≐\left[R_{i},\;p_{i},\;v_{i},\;b_{i}^{a},\;b_{i}^{g}\right]\in {\mathbb{R}}^{15},$$

where ***R***, ***p***, and ***v*** represent the IMU rotation, position, and velocity, respectively; ***b****^a^* and ***b****^g^* are the IMU accelerometer bias and gyroscope bias, respectively; Pose (***R***, ***p***) belongs to SE(3); and ***v***, ***b****^a^*, ***b****^g^* ∈ ℝ^3^.

**Figure 5:**
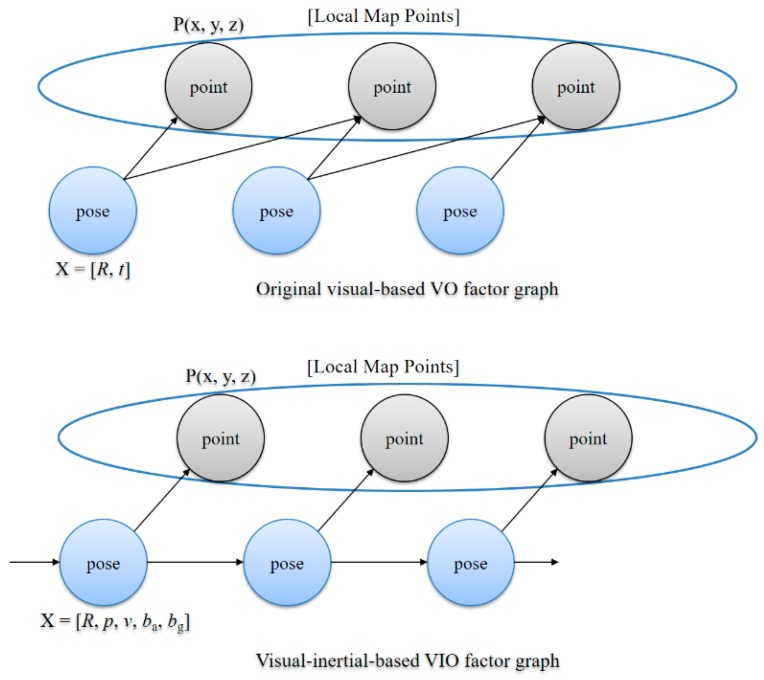
Comparison of the original visual-based VO factor graph and visual–inertial-based VIO factor graph.

In the bundle adjustment, we replace the existing 6-dimensional pose with a 15-dimensional pose and add the preintegrated IMU data as a constraint to the visual–inertial-based VIO factor graph as shown in Figure 5. We solve this optimization problem using the Levenberg–Marquardt algorithm [34] implemented in g2o [35].

### 3.2. Optical-Flow-Based Fast Visual Odometry

As described above, in the feature-based SLAM method, the feature points are extracted and the description is calculated for every frame. This operation is time consuming. For example, the ORB feature point extraction and description operations in ORB-SLAM require approximately 10 ms or more. Moreover, SLAM performs tasks such as feature point matching, pose calculation from matching feature points, and local map updating for each frame [2].

Therefore, we propose an optical-flow-based fast VO to quickly calculate the relative pose between the current frame and the previous frame. Using the IMU, the motion change between the two frames is calculated in the IMU preintegration step. The fast VO method is used for frames with relatively low significance and operations such as mapping of SLAM and creation of keyframes are omitted. This method reduces the computational complexity of SLAM, thereby rendering it suitable for the fast calculation of location information, such as in an AR application environment. Since the fast VO method is performed when the IMU preintegration value is small, it exhibits a better effect when the camera motion is relatively slow. 

**Figure 6:**
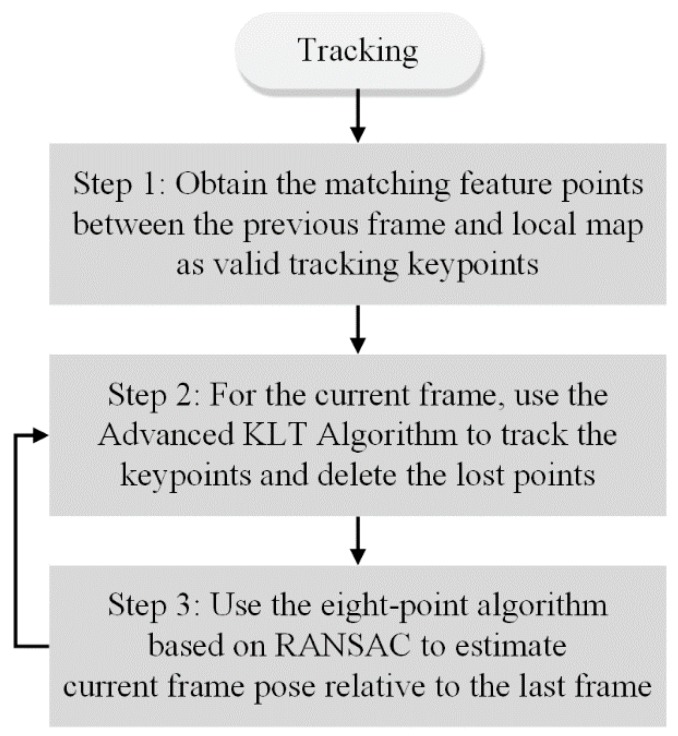
Flowchart of the optical-flow-based fast VO.

Figure 6 shows a flowchart of the optical-flow-based fast VO, which is divided into three steps.

In the first step, when tracking the optical-flow-based fast VO in the tracking module, matching feature points are acquired between the previous frame and the local map. These points are used as valid tracking keypoints and the previous frame is set as the reference frame. 

In the second step, a tracking operation based on an advanced Kanade-Lucas-Tomasi (KLT) feature tracking algorithm is performed on the current frame.

In the third step, the relative pose between two frames is calculated using the eight-point [8] algorithm each time using the matched keypoints. If the subsequent frame continues to perform the optical-flow-based fast VO, only steps 2 and 3 are executed.

#### 3.2.1. Advanced KLT Feature Tracking Algorithm

The optical flow method calculates the motion of a pixel between frames over time. When the camera moves, the position of the pixel in the frame also changes. An optical low method can track the motion of a pixel.

The calculation method for all the pixels is a dense optical flow and the calculation method for some pixels is a sparse optical flow. KLT [36] is a representative sparse optical flow algorithm for feature tracking. We use this method to track the feature points between consecutive frames.

**Figure 7:**
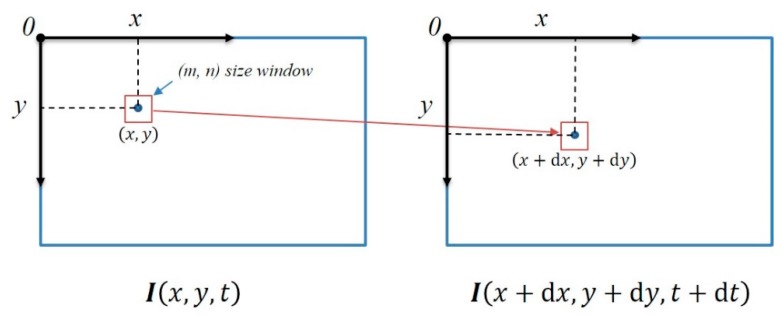
Diagram of the Kanade-Lucas-Tomasi (KLT) feature tracking algorithm.

The optical flow method assumes constant grayscale values. In other words, the grayscale value of the same spatial point pixel does not change over all the frames. As shown in Figure 7, when a pixel at position (*x*, *y*) at time *t* moves to position (*x* + d*x*, *y* + d*y*) at time *t* + d*t*, the grayscale value of the two pixels is the same.

According to the assumption of grayscale constant, the formula is

(8) I(x+dx, y+dy, t+dt)=I(x, y, t).

By performing Taylor expansion and retaining the first-order term, we obtain

(9) $$I\left(x+dx,\;y+dy,\;t+dt\right)\approx \;I\left(x,\;y,\;t\right)+\frac{\partial I}{\partial x}dx+\frac{\partial I}{\partial y}dy+\frac{\partial I}{\partial t}dt.$$

Substituting Equation (8) into Equation (9), we obtain

(10) $$\frac{\partial I}{\partial x}dx+\frac{\partial I}{\partial y}dy+\frac{\partial I}{\partial t}dt=0.$$

Dividing both sides by d*t*, we obtain the optical flow constraint equation:

(11) $$\frac{\partial I}{\partial x}\frac{dx}{dt}+\frac{\partial I}{\partial y}\frac{dy}{dt}=-\frac{\partial I}{\partial t},$$

where d*x*/d*t* is the motion velocity of the pixel along the *x*-axis, and d*y*/d*t* is the motion velocity along the *y*-axis, which are denoted as **u** and **v**, respectively. Further, ∂*I*/∂*x* is the gradient of the pixel in the *x*-direction and ∂*I*/∂*y* is the gradient of the pixel in the *y*-direction, which are denoted as ***I****_x_* and ***I****_y_*, respectively. Furthermore, ∂*I*/∂*t* is the change in the image grayscale over time, which is denoted as ***I****_t_*. The above expression can be written as

(12) $$\left[\begin{array}{cc} I_{x} & I_{y} \end{array}\right]\left[\begin{array}{c} u\\ v \end{array}\right]=-I_{t}.$$

The above equation is a linear equation with two variables, and cannot be solved only by one pixel. In the KLT method, it is assumed that the motion of the pixels in the window of size (*m*, *n*) around the pixel is the same. We obtain *m* × *n* number of equations

(13) $${\left[\begin{array}{cc} I_{x} & I_{y} \end{array}\right]}_{k}\left[\begin{array}{c} u\\ v \end{array}\right]=-I_{tk},\;k=1,\ldots ,mn.$$

***A*** and ***b*** are defined as follows:

(14) $$A=\left[\begin{array}{c} {\left[\begin{array}{cc} I_{x} & I_{y} \end{array}\right]}_{1}\\ \vdots \\ {\left[\begin{array}{cc} I_{x} & I_{y} \end{array}\right]}_{k} \end{array}\right],\;b=\left[\begin{array}{c} I_{t1}\\ \vdots \\ I_{tk} \end{array}\right].$$

Substituting Equation (14) into Equation (13), we obtain

(15) $$A\left[\begin{array}{c} u\\ v \end{array}\right]=-b.$$

In order to obtain the components *u* and *v* of the motion velocity of the pixel, the least-square solution of the overdetermined linear equation is calculated as follows:

(16) $${\left[\begin{array}{c} u\\ v \end{array}\right]}^{*}=-{\left(A^{T}A\right)}^{-1}A^{T}b.$$

Thus, we can obtain the motion vector of the pixel to calculate the position of the tracking keypoint in the current frame.

However, if the image resolution is large or the distance over which camera moving is far, the motion of the points in the window may be different, which will result in a larger computational error. Thus, we use the pyramid KLT algorithm to reduce the computational error, in order to improve the accuracy and robustness of the optical flow calculation. The pyramid KLT algorithm involves the detection of different resolution images—From the image with the smallest resolution at the top, gradually increase the resolution to the original image at the bottom.

In order to consider the effects of real-time problems, we use a three-layer pyramid. From the uppermost layer, the KLT algorithm is used to calculate the motion of the pixel, and after the scale transformation, it is used as the initial value of the subsequent layer; higher accuracy is calculated at the subsequent layer, and finally the motion of the pixel is calculated in the original image.

The optical flow method is very fast when a small number of tracking keypoints is used. For example, if the number is less than 100, the execution time is short and the efficiency is high. If 500 feature points per frame are used in tracking, there are approximately 100 matching points between two consecutive frames. Therefore, we use the keypoints obtained in step 1 to track the current frame, as shown in Figure 7, and subsequently delete any keypoint that fails to be tracked in the current frame. A keypoint that has been successfully tracked is stored with the current frame, and the current frame is set as the new reference frame. If the subsequent frame continues to execute the optical-flow-based fast VO, step 2 is immediately executed without executing step 1.

If the number of successfully tracked keypoints is less than the threshold, tracking fails. In this case, the current frame exits from optical-flow-based fast VO module and attempts to retrace itself back to the VIO module.

The optical flow method may not be accurate if the grayscale-invariance assumption is violated when the surrounding environment changes or the camera exposure parameter changes. Since the method proposed in this paper uses only a few consecutive frames each time for tracking, it is not significantly influenced by this problem.

#### 3.2.2. Pose Estimation

In optical-flow-based fast VO, the relative pose between two frames can be estimated in epipolar geometry [37] using 2D–2D image matching. In epipolar geometry, we generally estimate the essential matrix from the matching point and recover R and t from the essential matrix.

If tracking is successful in step 2, the matching point between the two frames can be obtained. We use the eight-point algorithm based on random sample consensus (RANSAC) [38] to estimate the essential matrix of two images.

**Figure 8:**
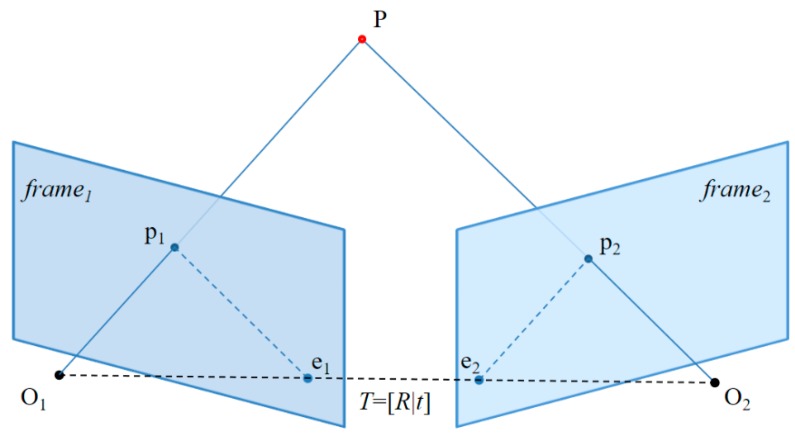
Diagram of the epipolar constraint.

Figure 8 illustrates the epipolar constraint. The projection of spatial point P at frame_1_ is *p*_1_ and the projection at frame_2_ is *p*_2_. ***K*** represents the intrinsic camera intrinsic parameters. The normalized coordinates are defined as follows:

(17) $$x_{1}=K^{-1}p_{1},\;x_{2}=K^{-1}p_{2}$$

where *x*_1_ and *x*_2_ satisfy the following homogeneous relation:

(18) $$x_{2}=Rx_{1}+t.$$

The epipolar constraint can be derived as follows:

(19) x2Tt^Rx1=0.

If the essential matrix ***E*** is defined as

(20) E=t^R,

We obtain

(21) $$x_{2}^{T}Ex_{1}=0.$$

In other words, the pose can be estimated by calculating the essential matrix ***E***. ***E*** is a 3 × 3 matrix with eight degrees of freedom (DoFs), and a constant factor. Therefore, the essential matrix ***E*** between two frames can be estimated using the eight-point algorithm based on RANSAC.

Subsequently, we can recover the camera rotation and translation matrix ***T*** = [***R***|***t***] from the estimated essential matrix ***E*** by using singular value decomposition (SVD) [39].

(22) $$\left(\begin{array}{c} u\\ v \end{array}\right)∼K\cdot [R|t]$$

Since the camera’s intrinsic parameters ***K*** are known, we can use the world-camera projection relationship in the above equation to calculate the position change (*u*, *v*) of the spatial point in the frame.

### 3.3. Adaptive Execution Module

In the adaptive execution module, the IMU preintegration value is used to predict the state of motion between the current frame and the previous frame. It is adaptively selected from VIO or optical-flow-based fast VO for current-frame tracking according to the state of motion.

In ORB-SLAM, each frame is compared with the local map. However, in a limited computing resource environment, the mapping thread may not be able to complete the task before the subsequent input frame arrives. Consequently, the local map information for tracking will not be ready and tracking fails, thus reducing the accuracy and robustness of the SLAM system.

ORB-SLAM will perform feature point extraction and tracking for each input image even if the camera is not moving. In other words, it performs unnecessary calculations and wastes computing power. This is a critical issue in environments with limited computing resources, such as mobile devices.

The goal of this module is to reduce the tracking time and reduce the demand for computing resources.

#### 3.3.1. Adaptive Selection Visual Odometry

**Figure 9:**
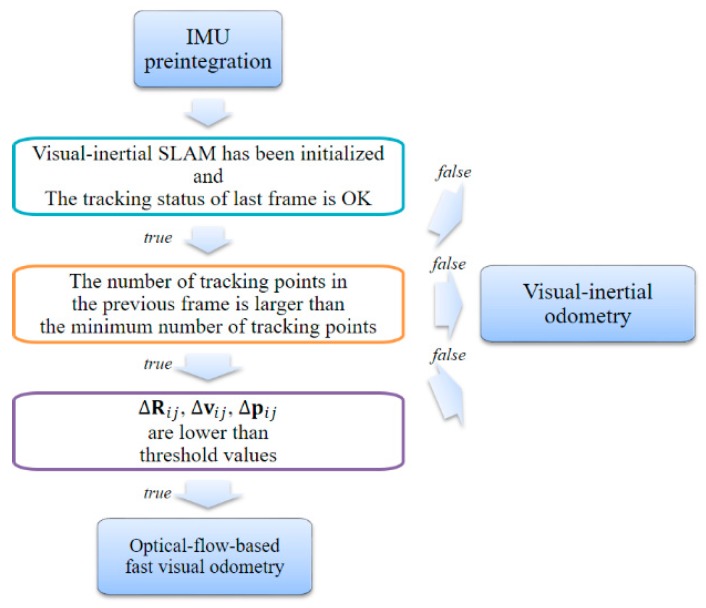
Flowchart of the adaptive execution module.

As shown in Figure 9, in the adaptive execution module, we first verify the system state and tracking state of the previous frame. Subsequently, we verify that the number of keypoints tracked in the previous frame satisfies the minimum number of keypoints required for tracking. Thereafter, the preprocessing data calculated by the IMU preintegration module are used as a condition for selecting an adaptive module. In Equation (3), we can calculate the changes in rotation, velocity, and position between the current frame and the previous frame, namely ΔR, Δv, and Δp, respectively. If the IMU measurement value is smaller than the set threshold value, the motion change is small. In other words, the parallax between the current frame and the previous frame is small. Finally, if all the conditions are satisfied, we execute the optical-flow-based fast VO module. Otherwise, we execute the VIO module.

#### 3.3.2. Adaptive Execution Policies

We also designed different levels of adaptive execution policies for different scenarios.

**Figure 10:**
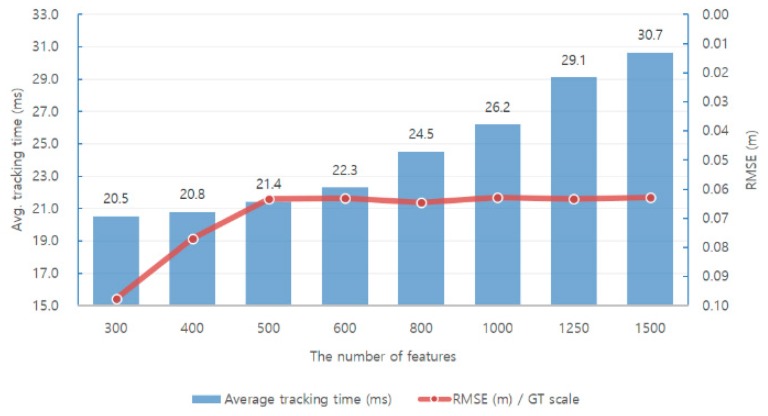
Average tracking time and the translation RMSE of the keyframe trajectory versus the number of features used in the monocular ORB-SLAM when running the EuRoC datasets.

**Figure 11:**
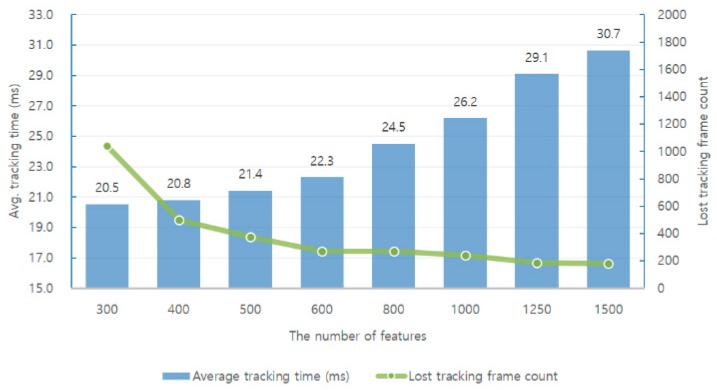
Average tracking time and the number of lost tracking frames versus the number of features used in the monocular ORB-SLAM when running the EuRoC datasets.

First, we analyzed the performance of the monocular ORB-SLAM via experiments. Figure 10 and Figure 11 show the relationships between performance, accuracy, and robustness when different numbers of features are applied in SLAM when running the EuRoC dataset [40].

A direct way to reduce the average tracking time is to reduce the number of features used in each image. Figure 10 and Figure 11 show that the average tracking time is reduced when the number of features is reduced. Simultaneously, the translation RMSE [41] value of the keyframe trajectory increases, and the number of lost tracking frames increases. There is a trade-off between the number of features used in the SLAM system and its accuracy and robustness.

An analysis of the results obtained using the EuRoC dataset shows that the accuracy and stability are significantly lowered when the number of features is less than 500. In order to ensure the efficiency of the computation, we use 500 features in the adaptive execution policy method. Subsequently, four modes are designed for additional execution policies, as listed in Table 2. The adaptive execution policies can be selected according to the performance of the mobile device in the system initialization step, or the user can preset the mode according to the application situation.

In addition to the optical-flow-based fast VO, several methods can be used to improve the performance of the adaptive execution module. Examples include reducing the local window size, modifying keyframe decisions and cullinlg policies, and reducing the number of DoFs of the pose in graph optimization.

In order to demonstrate the results more intuitively, we only use the proposed optimization methods.

## 4. Experiments and Results

In this section, we evaluate the proposed adaptive visual–inertial SLAM system focusing on two main goals. One is a comparison of the SLAM system using the proposed VIO with the existing ORB-SLAM system. The accuracy and robustness of the SLAM system are improved because the IMU measurement data is added to the pose optimization process. However, this requires additional operations and time. The other goal is to achieve real-time operation efficiency and accuracy of the visual–inertial SLAM system using the proposed adaptive execution module. The adaptive execution module improves the performance by dynamically applying the optical-flow-based fast VO in tracking.

**Figure 12:**
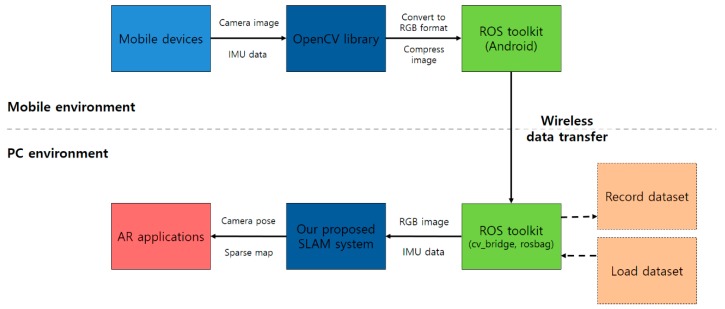
Structural architecture of the simulation.

We implemented and analyzed the proposed system in a PC environment. We compared the keyframe trajectory of the SLAM system with the ground-truth data and calculated the translation RMSE [42,43] of the keyframe trajectory for each sequence. We further analyzed the accuracy and the number of lost frames. Finally, we simulated the system using actual mobile device data by transmitting sensor data measured in real time from an Android smartphone to PC. The structural architecture of the simulation is shown in Figure 12. Our system only runs on a CPU and does not use any hardware acceleration methods.

Table 3 provides the specifications for the desktop, mobile devices, and development software environment. We experimented with the PC environment, and conducted experiments using the EuRoC dataset. The EuRoC dataset [40] has a total of 11 sequences and is recorded using a micro aerial vehicle. Sequences are measured in a machine hall and two different rooms, and classified into easy, medium, and difficult levels according to the illumination, texture, and motion speed. The EuRoC dataset provides stereo images, microelectromechanical systems IMU, ground-truth data, etc. The imaging system uses a global shutter camera, supports the size of 752 × 480, and the image data frequency of 20 FPS, and IMU data supports the data frequency of 200 Hz. The ground-truth data is used for comparison when analyzing the accuracy of the SLAM estimated trajectory. Therefore, the EuRoC dataset is widely used for benchmarking SLAM systems.

Moreover, we used the OpenCV 3.2.0 library [44] and the ROS indigo toolkits [45]. ROS indigo default support for OpenCV 2.4 version, but OpenCV 2.4 version and 3.2 version do not support simultaneous use. Therefore, we use the OpenCV 3.2 version to recompile the ROS indigo cv_bridge package.

### 4.1. Monocular ORB-SLAM Evaluation

We first analyzed the effect of the average tracking time, SLAM system accuracy and robustness when using features of different sizes in each frame in the monocular ORB-SLAM.

Table 4 provides the results of execution of all the sequences of the EuRoC dataset when features of different sizes are applied in the monocular ORB-SLAM. The results show the average tracking time, translation RMSE of the keyframe trajectory, and number of total lost tracking frames. The unit of measurement for the average tracking time is millisecond, and the unit of translation RMSE is meter.

If the RMSE value is small, the SLAM system accuracy is high. If the number of lost tracking frames is small, the robustness is high. If this value is more than a certain percentage of the total number of frames, it indicates that the tracking has failed. In the experimental results, the tracking failure case is marked as X.

The movement of the V2_03_difficult sequence is extreme; hence, when the feature size is set to less than 500, the tracking fails, and if it is set to 500, the number of lost tracking frames is 250.

At the end of the V2_01_easy sequence, the camera is covered by the object and the tracking is lost. The resulting data shows that the number of lost tracking frames is approximately 108. This is noise data which is not required to reflect the actual SLAM performance. Therefore, we removed the data from this part when evaluating the performance.

Figure 10 and Figure 11 show the relationship between the performance, accuracy, and robustness when different number of features are applied in SLAM when running the EuRoC dataset.

Experimental results show that increasing the number of features used in each frame increases the average tracking time and also increases the system accuracy and robustness accordingly. A direct way to reduce the average tracking time is to reduce the number of features used in each image. Simultaneously, the translation RMSE of the keyframe trajectory increases and the number of lost tracking frames also increases. There is a trade-off between the number of features used in the SLAM system and the accuracy and robustness. Moreover, if the experimental results are classified into three different levels and analyzed, the higher the level, the greater the influence on accuracy and robustness. Moreover, we can observe that the difficult level is significantly affected by the number of feature points.

An analysis of the results on the EuRoC dataset shows that the accuracy and stability are significantly lowered when the number of features is less than 500. In order to ensure the efficiency of the computation, we use 500 features in our proposed methods for testing.

### 4.2. Visual–Inertial Odometry SLAM Evaluation

We implemented the proposed VIO method and compared the visual–inertial SLAM with the monocular ORB-SLAM using the EuRoC dataset.

The accuracy of the result of initialization has a decisive influence on the accuracy of the entire SLAM system. In our proposed VIO method, we estimated the IMU biases and local 3D map scale at a certain amount of visual–inertial initialization time in the initialization step. We confirmed via the experiment that the visual–inertial initialization method is relatively stable, with a scale error typically less than 1.5% for the EuRoC dataset when the initialization time is set as 15 s.

**Figure 13:**
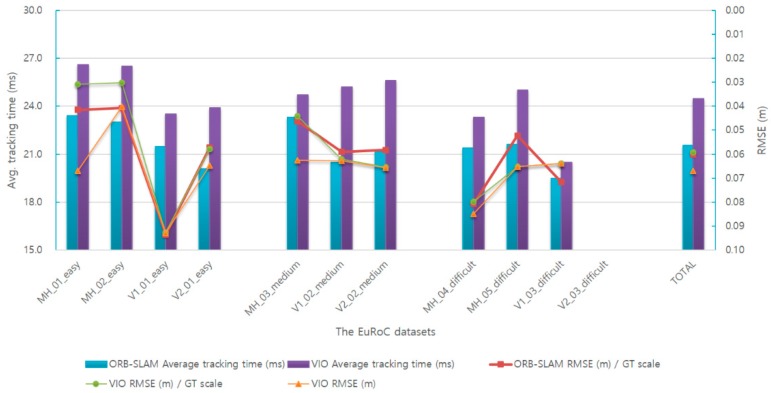
Evaluation of VIO-based SLAM and comparison with the monocular ORB-SLAM.

Table 5 and Figure 13 illustrate the comparison of the performances of the VIO-based SLAM and the monocular ORB-SLAM. The experimental results for the EuRoC dataset are shown in the same experimental environment with 500 features.

The existing monocular SLAM cannot calculate the true scale value. Therefore, to compare the keyframe trajectory with the ground-truth data, data preprocessing must be performed to match the ground-truth scale. The result of this comparison shows the accuracy of the monocular SLAM system, but it does not include scale information and therefore includes a scale error.

In order to compare the visual–inertial SLAM system with the monocular ORB-SLAM system, we divided the test results into the translation RMSE with GT scale and the translation RMSE without GT scale according to ground-truth scale matching.

Since the motion of the V2_03_difficult sequence is extreme, the proposed method and the monocular ORB-SLAM result in tracking failure.

Experimental results show that the average accuracy of the proposed VIO-based SLAM increases by 1.6% compared to the existing monocular ORB-SLAM, and the mean scale error of the proposed VIO-based SLAM is 0.58%. However, the average tracking time increases by 13.6% owing to the addition of IMU measurement data. The mean translation RMSE value is 0.067 m when the ground-truth scale matching is not applied to the estimated keyframe trajectory in the VIO-based SLAM, and is reduced by 13.2% compared to the translation RMSE value using the ground-truth scale matching. Simultaneously, the system robustness improves and shows good effects with a blurred image and pure rotation.

The following is the classification and analysis for different levels. At the easy level, the accuracy improves by 9.2% and the average tracking time increases by 14.2%. At the medium level, the accuracy reduces by 4.9% and the average tracking time increases by 16.2%. At the difficult levels, accuracy reduces by 2.1% and the average tracking time increases by 10.1%. Moreover, the system robustness increases at all levels.

When the proposed method is compared to the monocular SLAM, the accuracy is observed to slightly increase, because the accuracy of the IMU sensor currently used in mobile devices is lower than the accuracy of the camera sensor. The reason for the increase in the average tracking time in the VIO method is that IMU measurement data are additionally applied for pose optimization.

### 4.3. Adaptive Visual–Inertial Odometry SLAM Evaluation

First, the time required for tracking by the optical-flow-based fast VO method and the VIO method was measured and compared in the AVIO-based SLAM. Table 6 provides the average execution time of the two odometry methods of AVIO-based SLAM for the MH_01_easy sequence when the level 1 adaptive execution policy is applied.

Experimental results show that the optical-flow-based fast VO method is 3.85 times faster than the VIO method. In other words, the higher the proportion of the optical-flow-based fast VO among the total tracking, the faster the average tracking time.

**Figure 14:**
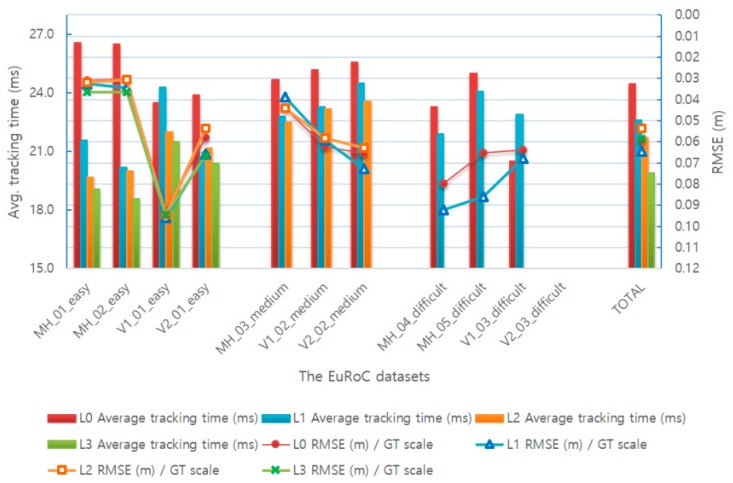
Comparison of the average tracking time and translation RMSE of keyframe trajectory with different level-sets of AVIO-based SLAM.

Table 7 and Figure 14 illustrate the experimental results for the proposed four adaptive execution policies. We can observe that the execution time can be significantly reduced by using the adaptive execution method. In the level 1 policy, the average tracking time is reduced by 7.8% compared to level 0, whereas the RMSE value increased by 8.5%. In the level 2 policy, the average tracking times are reduced by 17.5% and 8.3% for the easy dataset and medium dataset, respectively, compared to level 0. In the level 3 policy, the average tracking time is reduced by 18.8% for the easy dataset compared to level 0.

Moreover, the adaptive execution module also affects the other modules, such as mapping and optimization. Therefore, in order to analyze the impact of AVIO method on the entire SLAM system, we tested it in a single-core single-threaded environment. Furthermore, because the performance of this environment limits the SLAM system to run in real time, while leading to SLAM performance degradation.

**Figure 15:**
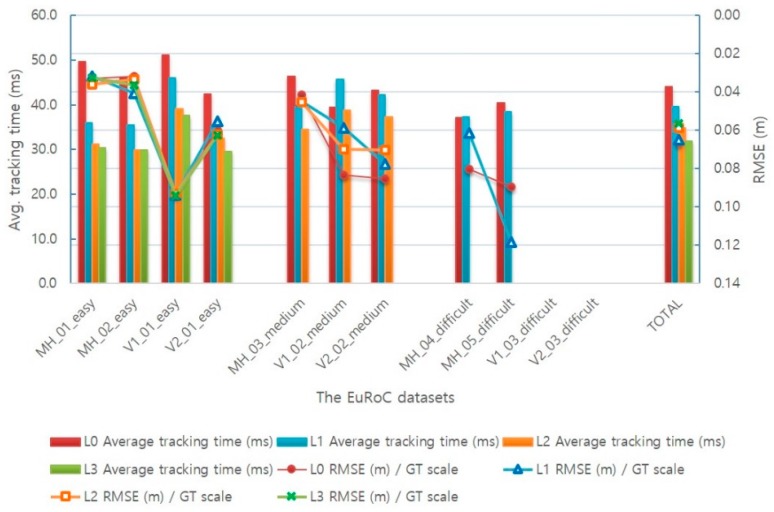
Comparison of the average tracking time and translation RMSE of keyframe trajectory with different level-sets of AVIO-based SLAM in a single-core single-threaded environment.

Table 8 and Figure 15 illustrate the experimental results for different level-sets of AVIO-based SLAM in a single-core single-threaded environment. The experimental results show that, in the level 1 policy, the average tracking time and RMSE value are reduced by 10.0% and 1.5% compared to level 0, respectively. In the level 2 policy, the average tracking times are reduced by 29.8% and 14.2% for the easy dataset and medium dataset, respectively, compared to level 0. In the level 3 policy, the average tracking time is reduced by 32.6% for the easy dataset compared to level 0.

### 4.4. Experiments of the Adaptive Visual–Inertial Odometry SLAM with Mobile Device Sensors

**Figure 16:**
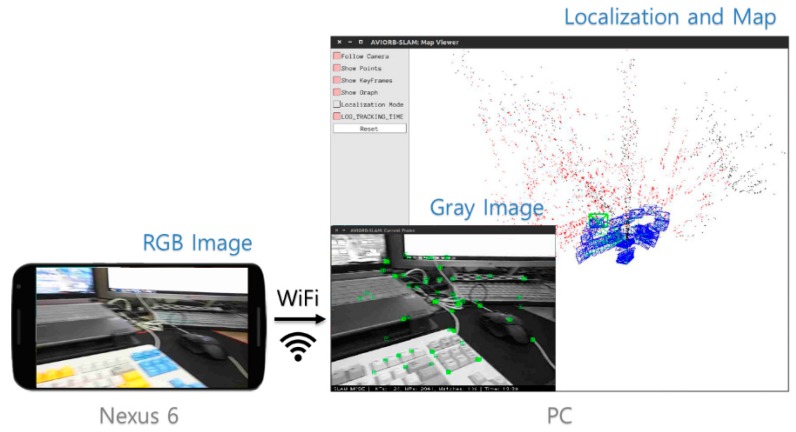
Screenshot of the proposed AVIO-based SLAM with real mobile device sensor data in a PC environment.

First, we use the OpenCV library to resize and convert the acquired camera image to RGB format on an Android smartphone. The image data and IMU data are transferred to the PC in real time under WiFi connection using the ROS toolkit. We obtained a 15 fps image of size 640 × 480 and 100 Hz IMU data on a Nexus 6 smartphone. After the camera-IMU calibration, the proposed method was tested in a PC environment. Figure 16 shows a screenshot of the proposed AVIO-based SLAM with real mobile device sensor data in a PC environment.

When we tested the proposed system using actual mobile device sensor data, the average tracking time was 23.2 ms. However, since the smartphone used in the experiment uses a low-priced rolling shutter camera and the image frequency is relatively low, moving it slightly faster will cause the image to be blurred and tracking to be lost. Although it can be solved to some extent with the VIO method, this system still has not achieved the desired robustness.

## 5. Conclusions

In this paper, we presented an adaptive monocular visual–inertial SLAM for real-time AR applications in mobile devices. First, we designed a VIO method that combines a camera and IMU sensor. Second, to support real-time faster tracking of AR applications in a mobile device environment, an optical-flow-based fast VO module was designed and combined with the existing ORB-SLAM system. Finally, we present an adaptive execution method that adaptively selected the tracking module according to the change in the IMU sensor value.

Experimental results show that the proposed technique achieves up to 18.8% of performance improvement, while reducing the accuracy slightly. Therefore, our methods can be adapted to improve the performance of SLAM for real-time AR applications in mobile devices.

## Figures and Tables

**Table 1:** Table of abbreviations.

Abbreviations	Definition
IMU	Inertial measurement units
VO	Visual odometry
VIO	Visual–inertial odometry
AVIO	Adaptive visual–inertial odometry
RMSE	Root-mean-square error
GT scale	Match the ground-truth scale
ROS	Robot operating system

**Table 2:** Table of adaptive execution policies.

Mode	Modules	Characteristics
Level 0	Visual–inertial odometry SLAM	Accuracy priority, no adaptive module
Level 1	Visual–inertial odometry and optical-flow-based fast visual odometry	Balance, suitable for all scenes
Level 2	Visual–inertial odometry and optical-flow-based fast visual odometry	Speed priority, suitable for easy and medium scenes
Level 3	Visual–inertial odometry and optical-flow-based fast visual odometry	Speed priority, suitable for only easy scenes

**Table 3:** Experiment environments.

**Desktop Specification**	
CPU	Intel Core i7-6700K
RAM	SDRAM 16 GB
OS	Ubuntu 14.04
**Phone Specification**	
Model name	Nexus 6
Android version	7.0 Nougat
**Development Software Environment**	
OpenCV	3.2.0 version
ROS version	ROS Indigo
Benchmark dataset	EuRoC dataset

**Table 4:** Average tracking time, translation RMSE of the keyframe trajectory, and number of lost tracking frames versus the number of features used in the monocular ORB-SLAM with EuRoC dataset.

Feature	MH_01_e	MH_02_e	V1_01_e	V2_01_e	MH_03_m	V1_02_m	V2_02_m	MH_04_d	MH_05_d	V1_03_d	V2_03_d
300	22.8	22.3	20.5	19.0	22.0	19.2	19.5	21.5	20.9	17.7	X
	0.057	0.052	0.093	0.057	0.064	0.069	0.087	0.216	0.150	0.131	X
	220	0	72	114	63	25	68	263	225	99	X
400	23.2	22.1	20.8	19.9	22.5	19.9	19.7	20.9	21.1	18.0	X
	0.043	0.047	0.094	0.060	0.045	0.065	0.071	0.147	0.054	0.146	X
	16	0	0	110	62	0	67	261	26	64	X
500	23.4	23.0	21.5	20.1	23.3	20.5	21.2	21.4	21.6	19.5	20.0
	0.042	0.041	0.094	0.057	0.046	0.059	0.058	0.081	0.052	0.072	0.095
	0	0	0	109	0	0	67	0	0	59	250
600	25.1	24.6	23.1	21.0	23.6	21.3	22.7	21.9	22.1	20.6	19.2
	0.046	0.037	0.095	0.059	0.039	0.064	0.056	0.076	0.052	0.064	0.104
	0	0	0	109	0	0	67	0	0	58	146
800	27.8	26.0	26.2	22.4	25.5	23.3	26.0	23.2	23.6	22.3	23.3
	0.043	0.036	0.097	0.057	0.041	0.064	0.056	0.087	0.068	0.064	0.097
	0	0	0	108	0	0	67	0	0	58	143
1000	29.5	28.4	28.8	24.2	27.2	25.2	27.4	25.6	24.5	23.0	24.3
	0.046	0.036	0.095	0.060	0.038	0.063	0.058	0.056	0.052	0.066	0.123
	0	0	0	108	0	0	0	0	0	117	121
1250	32.3	30.9	32.2	27.9	29.5	27.7	31.1	27.8	27.5	26.5	26.7
	0.044	0.035	0.095	0.058	0.039	0.064	0.058	0.062	0.050	0.071	0.119
	0	0	0	108	0	0	0	0	0	52	136
1500	33.5	32.9	33.6	29.4	31.4	29.7	32.0	29.3	29.3	27.8	28.3
	0.044	0.035	0.096	0.056	0.038	0.064	0.057	0.051	0.050	0.071	0.128
	0	0	0	108	0	0	0	0	0	55	127

**Table 5:** Evaluation of VIO-based SLAM and comparison with the monocular ORB-SLAM.

	ORB-SLAM (500)			VIO-SLAM (500)				
Dataset	ORB Average Tracking Time (ms)	ORB RMSE (m)/GT Scale	Lost Tracking Frame Count	VIO Average Tracking Time (ms)	VIO RMSE (m)/GT Scale	Scale Error	VIO RMSE (m)	Lost Tracking Frame Count
MH_01_easy	23.4	0.042	0	26.6	0.031	1.3%	0.067	0
MH_02_easy	23.0	0.041	0	26.5	0.030	0.6%	0.040	0
V1_01_easy	21.5	0.094	0	23.5	0.093	0.3%	0.093	0
V2_01_easy	20.1	0.057	109	23.9	0.058	1.2%	0.065	108
**Easy level**	**22.0**	**0.058**	**109**	**25.1**	**0.053**	**0.86%**	**0.066**	**108**
MH_03_medium	23.3	0.046	0	24.7	0.044	1.1%	0.063	0
V1_02_medium	20.5	0.059	0	25.2	0.062	0.5%	0.063	0
V2_02_medium	21.2	0.058	67	25.6	0.066	0.2%	0.066	0
**Medium level**	**21.7**	**0.055**	**67**	**25.2**	**0.057**	**0.61%**	**0.064**	**0**
MH_04_difficult	21.4	0.081	0	23.3	0.080	0.4%	0.085	0
MH_05_difficult	21.6	0.052	0	25.0	0.065	0%	0.065	0
V1_03_difficult	19.5	0.072	59	20.5	0.064	0.1%	0.064	0
V2_03_difficult	X	X	X	X	X	X	X	X
**Difficult level**	**20.8**	**0.068**	**59**	**22.9**	**0.070**	**0.18%**	**0.071**	**0**
Total	21.6	0.060	235	24.5	0.059	0.58%	0.067	108

**Table 6:** Comparison of the average execution time between optical-flow-based fast VO method and VIO method.

Optical-Flow-Based Fast Visual Odometry	Mean Time (ms)	Visual–Inertial Odometry	Mean Time (ms)
Obtain keypoints	0.16	ORB extraction	20.85
Advanced KLT tracking	2.53	Initial Pose Estimation with IMU	5.09
Pose Estimation	6.13	TrackLocalMap with IMU	8.02
**Total**	**8.82**		**33.96**

**Table 7:** Evaluation of AVIO-based SLAM.

	VIO (500)-L0		AVIO-L1 (Suitable for All)		AVIO-L2 (Suitable for Easy & Medium)		AVIO-L3 (Suitable for Easy)	
Dataset	Level 0 Average Tracking Time (ms)	Level 0 RMSE (m)/GT Scale	Level 1 Average Tracking Time (ms)	Level 1 RMSE (m)/GT Scale	Level 2 Average Tracking Time (ms)	Level 2 RMSE (m)/GT Scale	Level 3 Average Tracking Time (ms)	Level 3 RMSE (m)/GT Scale
MH_01_easy	26.6	0.031	21.6	0.032	19.7	0.032	19.1	0.036
MH_02_easy	26.5	0.030	20.2	0.034	20.0	0.031	18.6	0.036
V1_01_easy	23.5	0.093	24.3	0.096	22.0	0.094	21.5	0.094
V2_01_easy	23.9	0.058	20.7	0.066	21.2	0.054	20.4	0.066
**Easy level**	**25.1**	**0.053**	**21.7**	**0.057**	**20.7**	**0.053**	**19.9**	**0.058**
MH_03_medium	24.7	0.044	22.8	0.039	22.5	0.044	X	X
V1_02_medium	25.2	0.062	23.3	0.059	23.2	0.058	X	X
V2_02_medium	25.6	0.066	24.5	0.073	23.6	0.063	X	X
**Medium level**	**25.2**	**0.057**	**23.5**	**0.057**	**23.1**	**0.055**	X	X
MH_04_difficult	23.3	0.080	21.9	0.092	X	X	X	X
MH_05_difficult	25.0	0.065	24.1	0.086	X	X	X	X
V1_03_difficult	20.5	0.064	22.9	0.068	X	X	X	X
V2_03_difficult	X	X	X	X	X	X	X	X
**Difficult level**	**22.9**	**0.070**	**23.0**	**0.082**	X	X	X	X
**Total**	**24.5**	**0.059**	**22.6**	**0.064**	**21.9**	**0.054**	**19.9**	**0.058**

**Table 8:** Evaluation of AVIO-based SLAM in a single-core single-threaded environment.

	VIO (500)-L0		AVIO-L1 (Suitable for All)		AVIO-L2 (Suitable for Easy & Medium)		AVIO-L3 (SUITABLe for Easy)	
Dataset	Level 0 Average Tracking Time (ms)	Level 0 RMSE (m)/GT Scale	Level 1 Average Tracking Time (ms)	Level 1 RMSE (m)/GT Scale	Level 2 Average Tracking Time (ms)	Level 2 RMSE (m)/GT Scale	Level 3 Average Tracking Time (ms)	Level 3 RMSE (m)/GT Scale
MH_01_easy	49.6	0.033	36.0	0.032	31.3	0.036	30.4	0.032
MH_02_easy	46.1	0.032	35.5	0.041	29.9	0.033	29.9	0.036
V1_01_easy	51.1	0.093	46.0	0.094	39.1	0.093	37.6	0.094
V2_01_easy	42.5	0.060	35.3	0.055	32.5	0.062	29.5	0.062
**Easy level**	**47.3**	**0.054**	**38.2**	**0.055**	**33.2**	**0.056**	**31.9**	**0.056**
MH_03_medium	46.4	0.041	39.7	0.045	34.5	0.045	X	X
V1_02_medium	39.4	0.083	45.8	0.059	38.8	0.070	X	X
V2_02_medium	43.3	0.085	42.3	0.077	37.3	0.070	X	X
**Medium level**	**43.0**	**0.070**	**42.6**	**0.060**	**36.9**	**0.062**	X	X
MH_04_difficult	37.2	0.080	37.4	0.061	X	X	X	X
MH_05_difficult	40.4	0.089	38.5	0.118	X	X	X	X
V1_03_difficult	X	X	X	X	X	X	X	X
V2_03_difficult	X	X	X	X	X	X	X	X
**Difficult level**	**38.8**	**0.085**	**38.0**	**0.090**	X	X	X	X
**Total**	**44.0**	**0.066**	**39.6**	**0.065**	**34.8**	**0.059**	**31.9**	**0.056**
